# Association between graded subchorionic hematoma and adverse pregnancy outcomes in singleton pregnancies: a prospective observational cohort study

**DOI:** 10.1007/s00404-023-06943-8

**Published:** 2023-02-23

**Authors:** Weizhang Liang, Xi Yan, Yifu Shi, Bingjun Chen, Luwan An, Bei Huang, Fang He

**Affiliations:** Department of Obstetrics and Gynecology, Guangdong Provincial Key Laboratory of Major Obstetric Diseases, The Third Affiliated Hospital of Guangzhou Medical University, Guangzhou, China

**Keywords:** Subchorionic hematoma, Singleton pregnancies, Adverse pregnancy outcomes, Miscarriage, Fetal growth restriction

## Abstract

**Objective:**

To investigate whether different grades of subchorionic hematoma (SCH) are involved in the timing of birth and the development of adverse pregnancy outcomes in singleton pregnant women.

**Methods:**

A total of 171 women with singleton pregnancies, 72 of whom had SCH before 20 weeks and between 12 and 20 weeks of gestational age (GA), were included in this study conducted between January 2018 and December 2021. These patients were divided into three subgroups based on the size of the subchorionic hematoma on ultrasound imaging. Baseline demographic data, obstetric outcomes, and risk factors for subchorionic hematoma were compared for the two groups.

**Results:**

A higher number of pregnancies from the SCH group resulted in miscarriage (30.56% versus 2.02%, *p* < 0.0001), early preterm birth (8.33% versus 1.01%, *p* = 0.0035), premature rupture of membranes (15.28% versus 4.04%, *p* = 0.0103), fetal growth restriction (9.72% versus 0%, *p* = 0.0015), and delivery 13.18 days earlier (274.34 ± 11.25 versus 261.16 ± 29.80, *p* = 0.0013) than those from the control group. Compared with SCH detected before 12 weeks of GA, the rate of miscarriage increased, and the live birth rate decreased significantly in patients with SCH caught between 12 and 20 weeks of GA. With the increase in hematoma size, the likelihood of miscarriage increased significantly. Further analysis found that delivery occurred earlier in the medium/large SCH group (271.49 ± 23.61 versus 253.28 ± 40.68/261.77 ± 22.11, *p* = 0.0004/0.0073) but not in the small SCH group (274.34 ± 11.25 versus 267.85 ± 21.01, *p* = 0.2681) compared to the control group. Our results also showed that the anterior placenta (52.04% versus 33.33%, p = 0.0005, OR = 0.3137, 95% CI [0.1585, 0.601]) is a protective factor for subchorionic hematoma.

**Conclusion:**

Our study shows that women with SCH are at a higher risk of adverse pregnancy outcomes and are independently associated with miscarriage, early preterm birth, premature rupture of membranes, and fetal growth restriction. A subchorionic hematoma, especially detected between 12 and 20 weeks of GA, is very likely to cause miscarriage or preterm birth in women with a medium or large subchorionic hematoma.

## What does this study adds to the clinical work

**Table:** 

The clinical significance of subchorionic hematoma (SCH) remains controversial in the past. This study showed that in women with singleton pregnancies, SCH is associated with an increased rate of miscarriage and is independently associated with early preterm birth, premature rupture of membranes, and fetal growth restriction.	

.

## Introduction

A subchorionic hematoma (SCH) is a hypoechogenic area surrounding the gestational sac observed on ultrasound, and it was first described in patients with symptoms of threatened miscarriage in 1981. A subchorionic hematoma is the pooling of blood between the chorionic membrane and the uterine wall, and it is caused by the separation of the chorion from the endometrium [1–3]. The reported incidence varies between 0.46% and 39.5% [4, 5], whereas it may rise to 30% in pregnant women with symptoms of threatened miscarriage [4, 6–9]. Previous studies also found that SCH is associated with an increased risk of miscarriage, preterm birth (PTB), placental abruption, preterm premature rupture of membranes [10], low birth weight, and fetal growth restriction (FGR) [11, 12]. However, some investigators have considered that there is no effect on miscarriage, preterm birth [13, 14], or mode of delivery [10] in later pregnancy. The clinical significance of subchorionic hematoma remains controversial.

The size of the SCH might affect pregnancy outcomes. The relationship between hematoma size and miscarriage was statistically significant in early pregnancy [15]. Large SCH (the ratio of the largest linear diameter of SCH to the linear diameter of the pregnancy sac, ratio > 1/2) before 12 weeks of GA has shown an increased risk of adverse pregnancy outcomes [16]. Seventy-eight percent of patients with symptoms of threatened miscarriage before 20 weeks had SCH less than 5 cm, whereas 21% had SCH more than 5 cm [9]. It has been reported that the mean size of hematoma before 20 weeks was 15 ml in miscarriage patients and 25 ml in premature delivery patients [17]. Mandruzzato et al. compared two groups of patients with SCH (volumes higher or lower than 15 mL before 13 weeks). They found an increased rate of miscarriage and premature delivery in the higher volume group [18]. In another ROC analysis, 56% of miscarriages occurred in patients with a volume of SCH greater than 32 ml before 12 weeks of gestation [19]. However, the SCHs diagnosed before 12 weeks of gestation will gradually disappear or become larger in later pregnancy. The SCHs diagnosed after 12 weeks of GA are usually larger and more harmful to the mother and fetus. Detecting an SCH on an early ultrasound is often distressing for patients. The exact impact on pregnancy outcomes between the different graded SCHs and an appropriate method to distinguish patients with adverse pregnancy outcomes is needed.

Thus, the objectives of this study were (1) to determine the association between the size of SCH and adverse pregnancy outcomes before 20 weeks of gestation and (2) to determine the risk factors for the development of SCH-affected pregnancies. The findings of this study would help provide information on the potential adverse outcomes for SCH patients.

## Methods

### Study design and participants

This study was conducted at the Third Affiliated Hospital of Guangzhou Medical College between January 2018 and December 2021. We performed a case–control analysis within a prospective observational cohort (FEPED cohort), including women with singleton pregnancies. The Ethics Committee of the hospitals approved the study protocol.

### Assessment of subchorionic hematoma

**Fig. 1:**
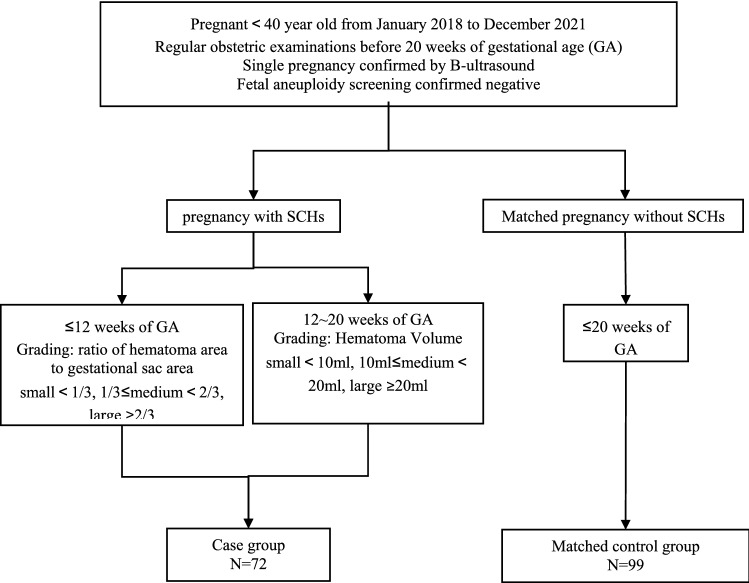
Recruitment profile of the case–control study. *SCH* subchorionic hematoma, *GA* gestational age. Pregnant women who had regular obstetric examinations before 20 weeks of gestational age (GA) were interviewed as potential candidates. The obstetric examinations included a routine physical examination and monitoring of fetal development. We recommend that all pregnant women choose at least two methods to screen for fetal aneuploidy: NT ultrasound, serum analyte screening, and noninvasive prenatal screening. Patients were excluded from the sample if they were over 40 years old, the obstetric examinations showed multiple pregnancies, abnormal NT ultrasound/serum analyte screening/noninvasive prenatal testing (NIPT), or the obstetric examination result was incomplete. The women diagnosed with SCHs were interviewed as potential candidates for the case group (SCH group). The women without SCHs (the control group) were included in the study as controls at a ratio of 1:1

Images from the ultrasound examinations, which were performed transvaginally, transabdominal, or both, were reviewed. The size of the SCHs was further graded according to the recommended method [15, 19], as shown in Fig. 1. The sonographic criterion for SCH in the first trimester was a crescent-shaped echo-free outline of the intact gestational sac. After 12 weeks of GA, there is usually an elongated echo-free area between the chorionic membrane and the uterine wall. Heller et al. [15] found that the semiquantitative method (hematoma size as a fraction of gestational sac size) was superior to other methods in assessing the size of a hematoma occurring in early pregnancy. However, this method is limited by needing to be applied and validated in the population after 12 weeks of GA. However, the semiquantitative method will become mathematically invalid after 12 weeks of GA compared with quantitative methods with three grades. Based on our early research, we improved the semiquantitative and quantitative methods to trichotomy for accurate analysis. Semiquantitative detection refers to calculating the ratio of subchorionic hematoma area to gestational sac area, with a small SCH comprising < 1/3 of the gestational sac, a medium SCH comprising 1/3–2/3 of the gestational sac, and a large SCH comprising ≥ 2/3 of the gestational sac. Quantitative detection refers to calculating the hematoma volume using the formula ($$volume=\frac{\mathrm{length}*\mathrm{width}*\mathrm{height}}{2}$$); the volume of a small SCH is less than 10 ml, the volume of a medium SCH is between 10 and 20 ml, and the volume of a large SCH is over 20 ml. The size of SCH was graded according to the semiquantitative method at the time of SCH diagnosis before 12 weeks of GA and according to the quantitative method between 12 and 20 weeks of GA. We also documented the location of the placenta and the size (length, width, height) of the hematoma for each participant.

Patients with SCH are generally recommended to review the size of the hematoma through ultrasound every 1 or 2 weeks in our center. SCH examination continues from the time of the hematoma’s first diagnosis until the hematoma disappears, and the final grade of diagnosis is based on the largest hematoma.

### Data collection and pregnancy outcomes

All participants underwent a series of routine obstetric examinations in our center, and the data obtained with patient consent included demographic data, placental location, mode of conception, and reproductive/gynecological/surgical history. All patients included in this study gave birth in our center, and pregnancy outcomes of the mother and fetus were followed up until postpartum. Miscarriage is a pregnancy loss before 28 weeks of gestation in China. Preterm birth (PTB) is defined as birth at < 37 weeks of gestation, and it is classified into early PTB (28 to < 32 weeks), moderate PTB (32 to < 34 weeks), and late PTB (34 to < 37 completed weeks of gestation). In addition, we also recorded pregnancy complications, including premature rupture of membranes (PROM), preterm premature rupture of membranes (PPROM), fetal growth restriction, fetal abnormalities, infection, and oligohydramnios. Oligohydramnios is defined as decreased amniotic fluid volume (AFV) for gestational age and diagnosed if an amniotic fluid index (AFI) shows a fluid level of less than 5 cm (or less than the 5th percentile), the absence of a fluid pocket 2 cm in depth, or a fluid volume of less than 300 mL at term.

### Statistical analysis

A two-tailed *t* test analyzed the association between pregnancy outcomes and SCH. Additional analyses included comparing the rate of each placental location and mode of conception between the SCH group and the control group using a simple chi-square test, as appropriate. Odds ratios (ORs) and their 95% CIs were calculated using the Newcombe–Wilson score method. All statistical analyses were undertaken using GraphPad Prism 8 software. *P* values less than 0.05 were considered statistically significant. Baseline characteristics of the two groups were described as the mean ± standard deviation for quantitative variables and as frequencies (%) for qualitative variables.

## Results

From January 2018 to December 2021, 72 SCH cases and 99 controls were included in this study (Fig. 1). Among those women, 36 were diagnosed with SCHs before 12 weeks of gestation, and 36 were diagnosed with SCHs between 12 and 20 weeks. Of those included, 27 had small SCHs, 26 had medium SCHs, and 19 had large SCHs. Before 12 weeks of GA, the probability of a small SCH (average diameter (mm): 12.77 [9.94, 15.59]) was 47.22% (17/36), of a medium SCH (average diameter (mm): 21.50 [16.99, 26.01]) was 27.78% (10/36), and of a large SCH (average diameter (mm): 34.50 [17.93, 51.07]) was 25.00% (9/36). Between 12 and 20 weeks of GA, the probability of a small SCH (average volume (ml): 4.21 [1.50, 8.60]) was 27.78% (10/36), of a medium SCH (average volume (ml): 16.45 [11.10, 20.00]) was 44.44% (16/36), and of a large SCH (average volume (ml): 79.68 [36.75, 170.00]) was 27.78% (10/36).

### Pregnancy outcomes

Overall, clinical characteristics and pregnancy outcomes between both groups are described in Table 1. Baseline characteristics were similar between the control and SCH groups, including age, height, weight (before pregnancy), BMI, pregnancy, and parity. Patients with chorionic hematoma had a higher rate of miscarriage before 20 weeks of gestation compared with the control group (16.67% (12/72) vs. 2.02% (2/99), *p* = 0.0010). In the SCH groups, 2 cases suffered a miscarriage in 20–24 weeks and 24–28 weeks, respectively. At the same time, no patients in the control group suffered a miscarriage after 20 weeks of pregnancy. At delivery, the weight gain in the SCH group was significantly lower than that in the control group (7.21 ± 7.55 vs. 13.44 ± 4.99, *p* < 0.0001). In addition, our results also showed that the rates of oligohydramnios (13.89% (10/72) vs. 1.01% (1/99), *p* = 0.0007), premature rupture of membranes (15.28% (11/72) vs. 4.04% (4/99), *p* = 0.0103) and FGR (9.72% (7/72) vs. 0% (0/99), *p* = 0.0015) were significantly increased during pregnancy in the SCH group. The overall rate of preterm birth (PTB) between 32 and 37 weeks of gestation was not statistically significant. However, the rate of early preterm birth (PTB) between 28 and 32 weeks of GA was 1.01% (1 of 99) in the control group, which was significantly lower (*P* = 0.0035) than that in the SCH group (9.72% (6 of 72)) (Table 1). Overall, the time of live birth in the SCH group was 44.88 days earlier than in the control group (271.49 ± 23.61 vs. 226.61 ± 61.19, *p* < 0.0001).

**Table 1:** Clinical characteristics and pregnancy outcomes of cases and controls

Parameter	Control group	SCH group	*p* value
Age(year), mean ± SD	31.18 ± 4.02	30.94 ± 4.00	0.7107
Height(cm), mean ± SD	159.26 ± 5.62	159.00 ± 4.94	0.7229
Weight(kg), mean ± SD	54.41 ± 9.22	53.97 ± 9.04	0.7229
Gestational weight Gain(kg), mean ± SD	13.44 ± 4.99	7.21 ± 7.55	< 0.0001
BMI (kg/m^2^), mean ± SD	22 ± 5.86	21.17 ± 3.68	0.4464
Pregnancy	2.17 ± 1.19	2.33 ± 1.29	0.5056
Parity	0.89 ± 0.94	0.96 ± 0.83	0.6821
Preterm birth*, n (%)	9(9.10)	12(16.67)	0.1603
Early PTB	0(0.00)	6(8.33)	0.0035
Moderate PTB	1(1.01)	1(1.39)	0.8201
Late PTB	8(8.08)	5(6.94)	0.7819
Miscarriage, *n* (%)	4(4.04)	22(30.56)	< 0.0001
Spontaneous miscarriage	2(2.02)	16(22.22)	< 0.0001
< 20w	2(2.02)	12(16.67)	0.0010
20–24w	0(0.00)	2(2.78)	0.0953
24–28w	0(0.00)	2(2.78)	0.0953
Elective abortion	2(2.02)	6(8.33)	0.0536
Live Birth, *n* (%)	95(95.96)	50(69.44)	< 0.0001
Vaginal delivery	58(58.59)	28(38.89)	0.0133
Cesarean section	37(37.37)	22(30.56)	0.1937
Obstetric complications, *n* (%)			
Oligohydramnios**	1(1.01)	10(13.89)	0.0007
PROM	4(4.04)	11(15.28)	0.0103
PPROM	0(0.00)	1(1.39)	0.2396
Infection	1(1.01)	2(2.78)	0.3847
FGR	0(0.00)	7(9.72)	0.0015
Fetal abnormalities	2(2.02)	3(4.17)	0.4108

^*^Preterm birth: defined as birth at < 37 weeks of gestation, and it is classified into early PTB (< 32 weeks), moderate PTB (32 to < 34 weeks), and late PTB (34 to < 37 completed weeks of gestation)

^**^Oligohydramnios: defined as decreased amniotic fluid volume (AFV) for gestational age and diagnosed if an amniotic fluid index (AFI) shows a fluid level of less than 5 cm (or less than the 5th percentile), the absence of a fluid pocket 2 cm in depth, or a fluid volume of less than 300 mL at term

*SCH* subchorionic hematoma, *BMI* body mass index, *PTB* preterm birth; PROM, premature rupture of membranes, *PPROM* preterm premature rupture of membranes, *FGR* fetal growth restriction

Further analysis of different SCH grades is described in Table 2. Compared with the control group, the rate of spontaneous miscarriage was significantly higher in women with small, medium, or large SCHs. Moreover, compared with SCH diagnosed before 12 weeks of gestation, the rate of spontaneous miscarriage increased significantly, and the live birth rate decreased significantly in patients diagnosed between 12 and 20 weeks of gestation. With the increase in hematoma size, the likelihood of miscarriage increased significantly, and the possibility of live birth decreased significantly. The premature birth rate increased dramatically in patients with medium and large hematomas, mainly occurring between 12 and 20 weeks of GA. However, no significant difference was found in the small and medium SCH groups (Table 2).

**Table 2:** Pregnancy outcomes of different graded SCH patients

Parameter, n (%)	Control group	small SCH	medium SCH	large SCH	p-value
Premature Birth	9(9.09)	2(7.41)	4(15.38)	5(26.32)	0.1388
≤ 12 weeks		1(5.88)	0(0)	1(11.11)	0.0447
12–20 weeks		1(10)	4(25.00)	4(40.00)	0.0955
Miscarriage	4(4.04)	7(25.93)	9(56.25)	6(31.58)	0.2419
≤ 12 weeks		2(11.76)	2(20.00)	1(11.11)	0.0581
12–20 weeks		5(50.00)	7(43.75)	5(50.00)	0.4226
Live Birth	95(95.96)	20(74.07)	17(65.38)	13(68.42)	0.2419
≤ 12 weeks		15(88.24)	8(80.00)	8(88.89)	0.0581
12–20 weeks		5(50.00)	9(56.26)	5(50.00)	0.4226

The *p*-value represents the comparison between small/medium/large SCH and the control group. It also means the effects of the different sizes between the three SCH groups detected at ≤ 20 weeks or between 12 and 20 weeks

*SCH* subchorionic hematoma

**Fig. 2:**
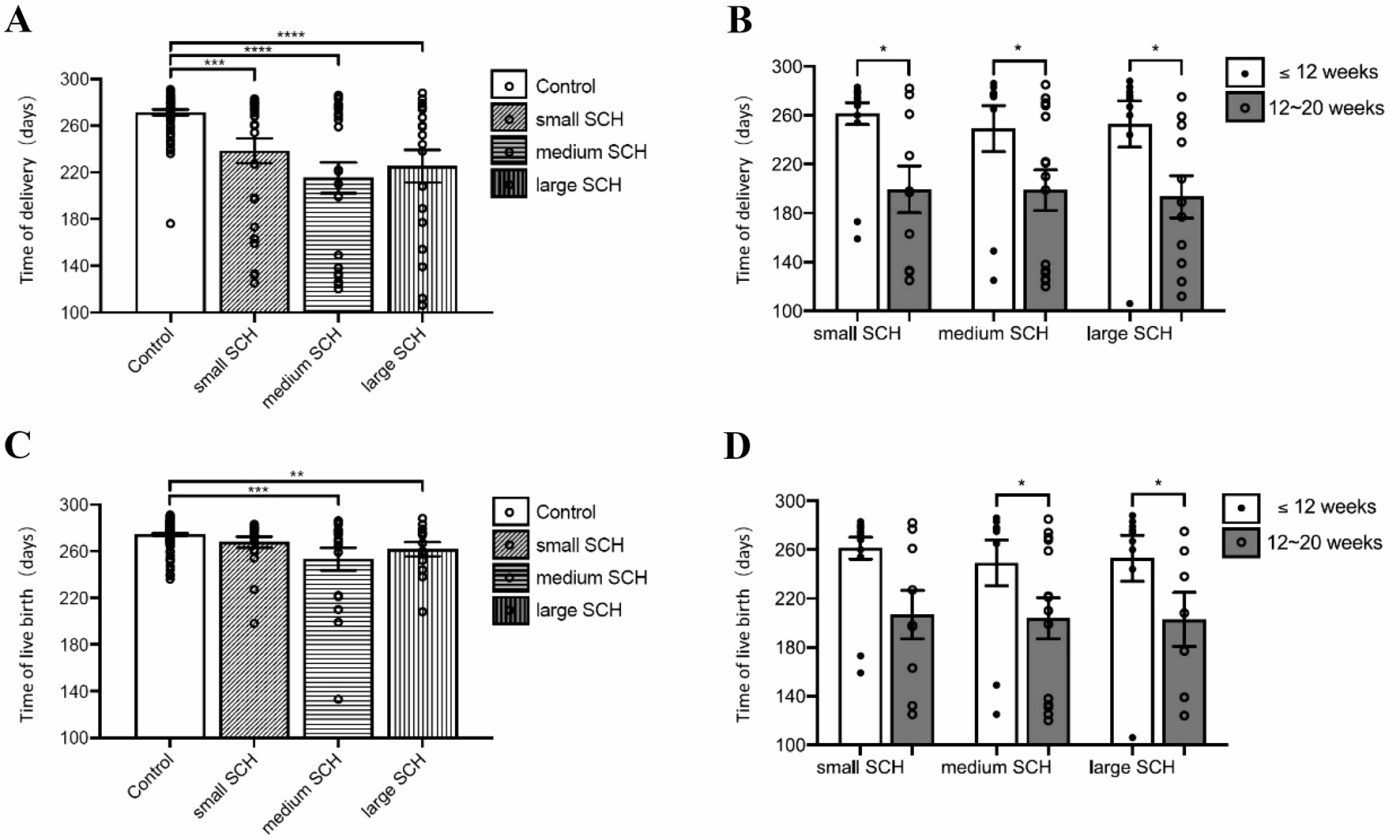
Time of delivery and live birth in patients with different subchorionic hematoma grades. **A** Comparison of the delivery time in patients with different SCH grades. **B** Comparison of the delivery time in SCH diagnosed before and after 12 weeks GA. **C** Comparison of the live birth time in patients with different SCH grades. **D** Comparison of the live birth time in SCH diagnosed before and after 12 weeks GA. *SCH* subchorionic hematoma. Statistical analysis was carried out using the Mann‒Whitney test. *p < 0.05; **p < 0.01; ***p < 0.001; ****p < 0.0001.

To determine whether the extent of SCH is associated with earlier delivery, we compared the gestational age at delivery/live birth between the graded SCH groups (Fig. 2). Patients without SCHs had more extended gestation periods than patients with small/medium/large SCH (271.49 ± 23.61 vs. 238.48 ± 55.12/215.27 ± 67.31/225.26 ± 60.74, *p* = 0.0001/ < 0.0001/ < 0.0001; Fig. 2A). Compared with SCH diagnosed before 12 weeks of GA, the gestation periods decreased significantly in patients diagnosed between 12 and 20 weeks of GA (255.92 ± 47.93 vs. 204.57 ± 58.9, *p* < 0.0001). The gestational ages at delivery in the two SCH groups (Fig. 2B) were 261.41 ± 36.78 and 206.89 ± 59.48 (*p* = 0.0279) in women with small SCH, 249.20 ± 59.65 and 203.93 ± 63.02 (*p* = 0.0229) in women with medium SCH, and 253.00 ± 56.69 and 202.86 ± 58.57 (*p* = 0.0418) in women with large SCH. Then, we excluded patients who had a miscarriage and compared the gestational age of live birth for the remaining patients. We found that women in the SCH group delivered 13.18 days earlier than those in the control group (274.34 ± 11.25 vs. 261.16 ± 29.80, *p* = 0.0013). Further analysis found that delivery occurred earlier in the medium/large SCH group (271.49 ± 23.61 vs. 253.28 ± 40.68/261.77 ± 22.11, *p* = 0.0004/0.0073) but not in the small SCH group (274.34 ± 11.25 vs. 267.85 ± 21.01, *p* = 0.2681) compared to the control group (Fig. 2C). Compared with SCH diagnosed before 12 weeks of GA, the gestational age decreased significantly in patients diagnosed between 12 and 20 weeks of GA (274.23 ± 9.51 vs. 246.58 ± 29.75, *p* = 0.0002). The gestational ages in the two SCH groups (Fig. 2D) were 274.13 ± 8.08 and 249.00 ± 35.71 (*p* = 0.2208) in women with small SCH, 277.25 ± 6.18 and 245.33 ± 32.09 (*p* = 0.0104) in women with medium SCH, and 271.38 ± 14.12 and 246.40 ± 25.26 (*p* = 0.0451) in women with large SCH.

### Risk factors for subchorionic hematoma

The distribution of modes of conception and placental locations in women between both groups is shown in Table 3. There was no statistically significant difference in the rate of different ways of conception. The placental location in the fundus, posterior wall, or lateral wall of the uterus was not different between the groups. Our study identified that women with singleton pregnancies with an anterior placenta are a protective factor for SCH [52.04% versus 33.33%, *p* = 0.0005, OR = 0.3137, 95% CI (0.1585, 0.601)].

**Table 3:** The risk factors for subchorionic hematoma between both groups

Risk Factors,* n* (%)	Control group (*n* = 99)	SCH group (*n* = 72)	OR [95%CI*]	*P* value
Mode of conception				
Natural conception	70(70.71)	58(80.56)	1.8480 [0.8848, 3.759]	0.1089
IVF-ET	23(23.23)	11(15.28)	0.5959 [0.2773, 1.283]	0.2456
Ovulation induction/artificial insemination	6(6.06)	3(4.17)	0.6739 [0.1800, 2.552]	0.7354
Placenta Location				
Uterine fundus	6(6.12)	5(9.26)	1.157 [0.3811, 4.161]	0.9999
Posterior wall of the uterus	32(32.65)	27(50.00)	1.256 [0.6613, 2.366]	0.5171
Anterior wall of the uterus	51(52.04)	18(33.33)	0.3137 [0.1585, 0.601]	0.0005
Sidewall of uterus	9(9.18)	4(7.41)	0.5882 [0.1941, 1.985]	0.5610
History				
Previous miscarriage	23(23.23)	22(30.56)	1.45 [0.7358, 2.859]	0.2966
Previous intrauterine operations	40(40.40)	39(54.17)	1.743 [0.9290, 3.169]	0.0882

^*SCH* subchorionic hematoma, *IVF-ET* in vitro fertilisation and embryo transfer^

^*^95% CIs were calculated using the Newcombe–Wilson score method

To understand whether the occurrence of SCH is related to previous spontaneous miscarriage, previous preterm births, and intrauterine procedures, we compared the distribution of patients who had miscarriages, preterm births, or intrauterine procedures between the two groups and calculated the OR value (Table 3). The incidence between the two groups and its OR value was 23.23% vs. 30.56% [*p* = 0.2966, OR = 1.45, 95% CI (0.7358, 2.859)] for previous spontaneous miscarriage and 40.40% vs. 54.17% [*p* = 0.0882, OR = 1.743, 95% CI (0.9290, 3.169)] for intrauterine procedures.

## Discussion

The prevalence of subchorionic hematoma did not differ by type of fertility treatment [20]. Prior studies in fertile populations also showed an increased rate of miscarriage [3, 10, 19]; however, other studies had a contrary opinion [6, 13, 14, 21]. This difference may be due to the different discovery times and grades of SCH; despite patients with a small SCH in early pregnancy, most of the SCH would not be detected in later pregnancy because of diminishing or absorption. In our cohort study, including spontaneous and IVF pregnancies of singleton pregnancies, we recruited patients with different grades of SCH found during early and middle pregnancy simultaneously. Our data suggest that SCH is associated with miscarriage and early PTB, and the rates of vaginal delivery, oligohydramnios, premature rupture of membranes (PROM), and fetal growth restriction are higher in patients with SCH. This finding is consistent with previous studies. Şükür and Tuuli highlighted a twofold to fourfold increased risk of SCH with spontaneous miscarriage compared to the non-SCH group [7, 10]. Although vaginal bleeding was observed in most patients, our study did not analyze it independently. Because vaginal bleeding is a subjective description, it has limitations in guiding clinical practice.

Most studies noted the association of very large hematomas with miscarriage (< 20 weeks). Our study was similar to previous studies; most miscarriages occurred before 20 weeks of gestation in patients with SCH. At the same time, a survey by Naert proved that SCH is not associated with any pregnancy outcomes at more than 20 weeks of gestation, including gestational age at delivery, preterm birth, birth weight, preeclampsia, placental abruption, and intrauterine fetal death at more than 20 weeks of gestation [13], which is inconsistent with our clinical observations. Our study also found that women with SCH had a higher rate of early PTB (< 32 weeks) delivered earlier than pregnant women without SCH after 37 weeks of gestation. Our finding is consistent with a retrospective cohort study conducted by Normal et al.[2] There is an increased risk of early PTB in SCH patients diagnosed before 22 weeks of GA. Peixoto et al.[22] demonstrated an equal rate of 16% preterm deliveries for both the SCH and non-SCH groups. However, their study was retrospective and only looked at SCH at one point. In contrast, our study was novel because it was a prospective observational cohort study and included a series of ultrasound examinations during gestation. In addition, Mantoni and Pedersen stated that the average hematoma size was 20 ml. There was no association between the hematoma size and the rate of miscarriage and premature birth in patients with or without SCH between 9 and 20 weeks [17]. In contrast, the patients with vaginal bleeding with a hematoma volume of fewer than 2 ml were excluded using the same evaluation methods of hematoma volume as us. In this study, patients with medium or large subchorionic hematomas had a statistically significant difference between a miscarriage and premature birth. Still, no difference was found between different sizes of hematomas. We also observed that small hematomas did not affect the pregnancy outcome, which may be related to the other evaluation methods of hematoma volume. Combined with our findings, these results indicate that there should be more focused counselling for highly anxious patients and providers. Patients with small SCHs can be reassured that there were no associated adverse maternal or obstetric outcomes. This may help counsel patients when an ultrasound detects a small SCH incidentally.

Furthermore, we found that 10 cases of oligohydramnios, 11 cases of premature rupture of membranes, and 7 cases of fetal growth restriction occurred in the SCH group. At the same time, few happened in the control group. SCH is believed to affect trophoblast invasion and impair changes in spiral arteries during pregnancy, thus leading to poor exchange of nutrients between the mother and fetus. This is consistent with the findings reported by Nagy et al. [11], which included 187 patients with SCH and 6488 controls, showing that the rate of fetal growth restriction (RR 2.4; CI 1.4, 4.1) was significantly higher in the SCH group. Similarly, in a study by Hashem et al. [12] in India, SCH detected by ultrasound in the first trimester of pregnancy was significantly associated with low gestational age at birth, low birth weight, low Apgar score at 1 and 5 min, and more NICU admissions.

Although most studies believe that the subchorionic hematoma is not related to the location of placental insertion, we found that the placental lying in the anterior wall of the uterus might be a protective factor for SCH. In contrast, Günay et al. [16] showed that hematomas in the anterior wall are more likely to cause placental abruption and early pregnancy loss. On the other hand, we found no difference between the control group and the SCH group regarding the modes of conception or reproductive, gynecological, and surgical history. A study by Peixoto et al. [22] found parity to be higher in patients with subchorionic hematoma. Similarly, Polish studies [23] by Truong et al. and Asato et al. found no differences in the rate of SCH in the IVF treatment cycle; however, Truong et al. found that they did not differentiate between fresh and frozen IVF [24], while Asato et al. found that SCH was more prevalent among frozen rather than new embryo transfer [25].

To our knowledge, the enlargement of the gestational sac increases with age and is even more noticeable after 12 weeks of gestation. However, the hematoma size does not increase in proportion to the size of the gestational sac. This ratio calculated using the semiquantitative method will become mathematically invalid and can yield very spurious results. Thus, we recommend using a quantitative method (volume of the SCH) to grade SCH in later pregnancy (after 12 weeks of GA).

The strengths of our study include multiple obstetric examinations of all participants throughout the pregnancy. This allows for monitoring the development of pregnancy outcomes of the different SCH grade populations. On this basis, SCH was stratified into small, medium, and large SCH; thus, it will help clarify the clinical importance of SCH in adverse pregnancy outcomes, such as a gestational week at delivery and birth weight, and delivery route. Subchorionic hematomas are usually located around the placenta and generally mature by 12 weeks of GA. The SCHs group was classified according to an appropriate measurement based on the time to diagnosis.

Our study also has several limitations. The evaluation of different graded SCH patients has limited statistical power due to these events’ rarity. SCH may have a low incidence and is rarely encountered in clinical practice in local areas. Thus, continued data collection and evaluation of pregnancies affected by SCH is necessary for better assessment. We relied on a sonographer inquiry of patient symptoms recorded in a categorical (yes/no) fashion, and we could not differentiate the extent of vaginal bleeding. In addition, we did not investigate whether aspirin or LMWH during conception or the 1st trimester affected vaginal bleeding or SCH. Clinical symptoms, duration of SCH, and drug abuse may be significant predictors of pregnancy outcome. In addition, our study may be limited using data from one obstetric center in China. The definition of miscarriage in this local study means the loss of an embryo or fetus before 28 weeks of pregnancy. In developed countries, miscarriage is a pregnancy loss before 20 or 24 weeks. The definition of miscarriage varies in different regions, and the threshold of embryo viability ranges from 20 to 28 weeks of pregnancy. This may overestimate the incidence and risk of miscarriage in our study. Thus, further multicenter studies should include larger populations. We also looked forwards to studying patients with clinical symptoms and drug abuse history to evaluate the associations with this.

In conclusion, in women with singleton pregnancies, SCH is associated with an increased rate of miscarriage and is independently associated with early preterm birth and fetal growth restriction. Future research is needed to examine the impact of these symptoms on SCH-affected pregnancies, with more attention to detail regarding the severity of symptoms.

## Data Availability

The data that support the findings of this study are available from the corresponding authors, upon reasonable request.
